# Diagnostic Challenges and Management Strategies of Pelvic Inflammatory Disease in Sexually Inactive Pediatric and Adolescent Patients: A Systematic Review of Case Reports

**DOI:** 10.3390/jcm14113971

**Published:** 2025-06-04

**Authors:** Adrian Surd, Rodica Mureșan, Andreea Oprea, Kriszta Snakovszki, Lucia Maria Sur, Lia-Oxana Usatiuc, Carmen-Iulia Ciongradi, Ioan Sârbu

**Affiliations:** 1 Pediatric Surgery and Orthopedics, “Iuliu Hațieganu” University of Medicine and Pharmacy, 400012 Cluj-Napoca, Romania; adisurd@elearn.umfcluj.ro; 2 Pediatric Surgery and Orthopedics, Emergency Children Hospital, 400370 Cluj-Napoca, Romania; muresanrodicaana@elearn.umfcluj.ro (R.M.); kriszta.sztrelenczuk@elearn.umfcluj.ro (K.S.); 3 Department of Pediatric Surgery, Emergency Childrens Hospital, 400370 Cluj-Napoca, Romania; oprea_andreea@elearn.umfcluj.ro; 4 Pediatrics, “Iuliu Hațieganu” University of Medicine and Pharmacy, 400217 Cluj-Napoca, Romania; sur.maria@umfcluj.ro; 5 Pathophysiology, Department of Functional Sciences, Faculty of Medicine, University of Medicine and Pharmacy “Iuliu Hațieganu”, 400349 Cluj-Napoca, Romania; 6 Pediatric Surgery and Orthopedics, “Grigore T. Popa” University of Medicine and Pharmacy, 700115 Iasi, Romania; carmen.ciongradi@umfiasi.ro (C.-I.C.); sarbu.ioan@umfiasi.ro (I.S.)

**Keywords:** pelvic inflammatory disease, non-sexually active, adolescent, case report, literature review

## Abstract

**Background and objectives:** Pelvic inflammatory disease (PID), primarily associated with sexually transmitted infections (STIs), represents a diagnostic challenge in virgin pediatric patients due to the often vague, non-specific symptomatology, which can mimic other conditions. Management prioritizes targeted antimicrobial therapy, with surgical intervention reserved for complications like tubo-ovarian abscess (TOA). The present systematic review aimed to critically evaluate the available evidence from case reports of PID in virgin pediatric and adolescent patients. **Methods:** The search strategy was in accordance with PRISMA guidelines. Case reports published up to March 2025 were searched through PubMed, Embase, Scopus, and Google Scholar databases. We included English-language case reports on non-sexually active pediatric and adolescent patients with available full text, excluding commentaries, reviews, and editorials. The Critical Appraisal Checklist for Case Reports was used for the quality assessment of case reports. Through descriptive analysis, PID symptoms, diagnostic, and management modalities were reviewed. The quality of the included case reports was assessed using the JBI Critical Appraisal Checklist. This review was not registered and did not receive external funding. **Results:** Among the 56 case reports searched, 20 reports were selected and analyzed based on eight criteria. The most frequently reported symptoms were lower abdominal pain (95.8%), fever (63.6%), and gastrointestinal symptoms (50%). Common comorbidities included urinary tract infections (22.7%), congenital anomalies (18.1%), and appendicitis history (18.1%). *Escherichia coli* and *Streptococcus* species were the predominant pathogens identified. All patients received antibiotic therapy, while 90.9% underwent surgical intervention. Favorable outcomes were achieved in 72.7% of cases, though 27.2% experienced complications or recurrences. **Conclusions:** Although commonly linked to sexual activity, PID should be considered in sexually inactive pediatric patients presenting with abdominal pain and adnexal masses. Early diagnosis, appropriate imaging, and timely treatment are crucial to improve outcomes and reduce complications. The evidence in this review is limited by its reliance on case reports, which may introduce bias and restrict generalizability.

## 1. Introduction

Pelvic inflammatory disease (PID) encompasses infection and inflammation of the female upper reproductive tract, including the uterus, fallopian tubes, ovaries, and adjacent pelvic structures. PID is uncommon among premenarchal and noncoital young women; however, several case reports have documented its occurrence in virginal females [1]. While PID can arise from various infectious agents, it is most commonly associated with sexually transmitted infections (STIs), particularly *Chlamydia trachomatis* and *Neisseria gonorrhoeae*, which ascend from the cervix, triggering an inflammatory cascade within the upper genital tract, thereby establishing PID as a significant complication of untreated STIs [2]. In sexually inactive patients, PID is best regarded as a polymicrobial infection, often involving organisms like *Escherichia coli*, *Streptococcus* species, anaerobes, and atypical bacteria, necessitating broad-spectrum antimicrobial therapy [3].

In these patients, PID may arise from alternative mechanisms such as hematogenous spread of pathogens, direct extension from adjacent infections (e.g., appendicitis and diverticulitis), or immune-mediated inflammatory responses [4]. Predisposing factors reported in this population include vaginal atresia, Hirschsprung disease, diabetes mellitus, and urinary tract anomalies, each contributing to increased vulnerability to ascending infections [5,6,7,8]. The clinical presentation of PID in this population is highly variable and often non-specific, often leading to delayed diagnosis and management. Lower abdominal pain is the most common symptom, frequently accompanied by fever, nausea, vomiting, dysuria, vaginal discharge, and menstrual irregularities. Given these overlapping features with other conditions such as appendicitis, ovarian torsion, and urinary tract infections, clinical diagnosis is complex [9].

Diagnostic approaches rely on a combination of clinical criteria, laboratory markers, and imaging modalities. Laboratory findings, including elevated inflammatory markers and leukocytosis, support the diagnosis. Following advanced imaging with ultrasound, CT, and MRI, a thorough differential diagnosis is essential to distinguish PID from pelvic tumors and other pathologies [1,2,10,11]. When uncertainty remains or complications like tubo-ovarian abscess are suspected, diagnostic laparoscopy serves as the gold standard, allowing for direct visualization, definitive diagnosis, and potential therapeutic intervention [12].

Given the typical association of PID with sexually transmitted infections, its presence in a non-sexually active child raises significant concern for sexual abuse. This underscores the need for clinicians to prioritize both the diagnostic evaluation of PID and the safeguarding of the patient through careful and sensitive assessment for abuse [13].

Pelvic inflammatory disease (PID) in pediatric patients carries substantial risks for both short- and long-term complications, including tubo-ovarian abscess recurrence, hydrosalpinx, sepsis, and chronic pelvic pain. Importantly, the potential for future infertility should be carefully considered, particularly in this young population. Structural damage resulting from inflammation and fallopian tube scarring may increase the risk of infertility or ectopic pregnancy. Furthermore, there is the possibility of iatrogenic infertility or subfertility, which may arise from invasive diagnostic or therapeutic interventions or from delayed treatment, leading to irreversible reproductive tract damage [14]. Early diagnosis, prompt antibiotic therapy, and individualized care strategies are critical to minimizing these adverse outcomes and preserving reproductive health in this vulnerable population.

This review aims to comprehensively examine the existing literature on PID in non-sexually active pediatric and adolescent patients by analyzing its epidemiology and evolving etiology, enhancing clinical awareness of PID in the differential diagnosis of abdominal pain and adnexal masses in this population, and emphasizing the importance of a coordinated, multidisciplinary approach to expedite diagnosis and facilitate timely intervention, thereby minimizing sequelae.

## 2. Materials and Methods

### 2.1. Search Strategy and Study Selection

**Figure 1:**
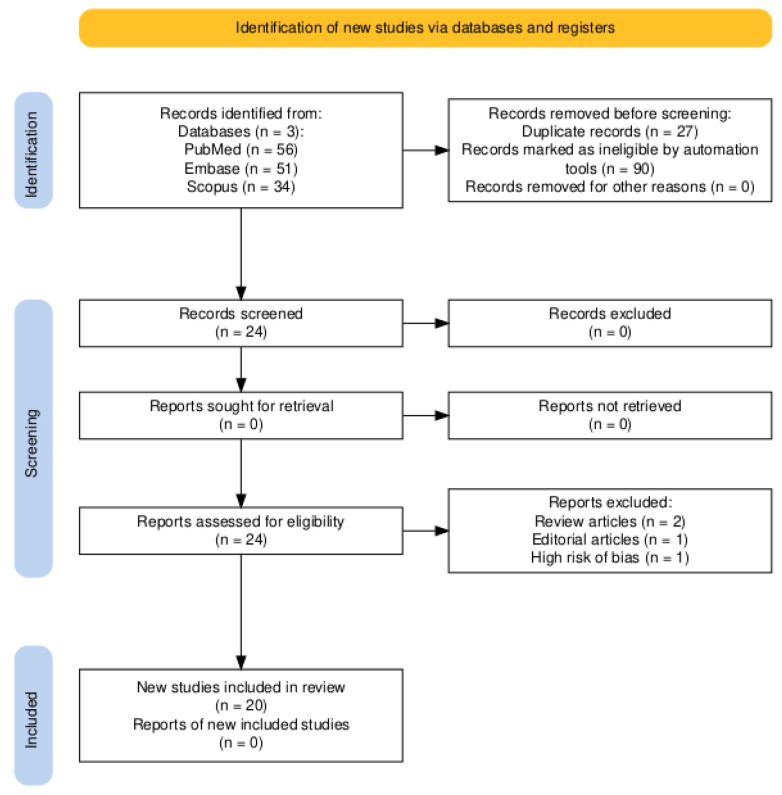
PRISMA flow diagram.

This systematic review followed the PRISMA 2020 guidelines [15]. The PRISMA flow diagram is shown in Figure 1. The review protocol was not registered in PROSPERO. 

A literature search was performed on four of the major databases, PubMed, Embase, Scopus, and Google Scholar databases, for case reports published up to March 2025. The search was performed according to the Boolean information retrieval method. Search terms included “pelvic inflammatory disease”, “tubo-ovarian abscess”, “non-sexually active”, “virgin”, “adolescent”, “pelvic abscess”, and “case report”. The title and abstract of all the studies were screened for relevancy. We also searched the bibliographies of all included studies to find additional studies that could be incorporated into our review. Duplicates were excluded using bibliographic citation management software (EndNote 20, Thomson Reuters, NY, USA).

### 2.2. Eligibility Criteria

We only included case reports involving pediatric and adolescent patients (under 18 years of age) who were confirmed to be sexually inactive where full-text articles were available. A thorough analysis of the articles was performed by two different investigators. The selection process was divided into two stages: the first stage focused on reading the titles and abstracts to verify the inclusion or exclusion criteria. The selected articles were moved on to the second stage, and by analyzing the contents of the articles, they were selected for further reviewing. For the second stage, all authors of this review were consulted to minimize the bias or any discrepancies. All articles that were selected after this two-stage process were included in our review.

### 2.3. Exclusion Criteria

Only articles published in English were included in this review. Commentary pieces, review articles, and editorials were excluded. Additionally, abstracts, conference proceedings, and other grey literature that lacked peer review or full-text access were excluded from the final analysis. Duplicate publications or multiple reports on the same case, unless they provided additional relevant information, were not included. Case reports that lacked sufficient clinical details or outcome data were not considered. Studies that did not focus on pediatric or adolescent patients—defined as individuals under 18 years of age—were excluded, as were reports involving sexually active individuals or those where the sexual-activity status of the patient was unclear. Articles without a confirmed clinical or radiological diagnosis of pelvic inflammatory disease (PID) were also excluded.

### 2.4. Risk of Bias Evaluation

The quality assessment of the systematic review of case reports was conducted using the Joanna Briggs Institute (JBI) Critical Appraisal Checklist for Case Reports [16]. Case reports meeting at least five of the eight criteria were considered to have acceptable quality. Consensus was reached among all researchers regarding the selected case reports. Each case report was independently assessed by multiple reviewers, and any discrepancies were resolved through discussion to reach consensus. Reports meeting at least five out of the eight criteria were deemed to have satisfactory methodological rigor. By systematically applying these criteria and involving multiple reviewers in the appraisal process, the likelihood of selection and reporting bias was reduced, enhancing the overall reliability of the findings in this review.

### 2.5. Data Collection

Data collected from the articles were systematically summarized in tables, which highlight the year of publication, primary authors, patient demographics (including age and relevant medical history), the total number of cases reported in each study, sexual history (including investigations for sexual activity or abuse), clinical presentation (specific symptoms and their characteristics), diagnostic methods (imaging, laboratory studies, and surgical findings), microbiology findings (specific pathogens isolated), and treatment approaches (including medical and surgical interventions).

We extracted all available results related to these outcomes, as reported in each study, regardless of time point or measurement tool. If multiple time points or tests were provided, we prioritized final outcomes at discharge or the last follow-up available. In cases of incomplete reporting, outcomes were recorded as “not reported”.

### 2.6. Data Analysis

Sensitivity analyses were conducted by excluding low-quality or atypical case reports to ensure the robustness of the synthesized findings. Certainty in the body of evidence was assessed qualitatively, considering consistency of findings across reports, clarity of clinical detail, and risk of reporting bias. Due to the inherent variability in case reports—including differences in study design, participant health conditions, intervention protocols, testing methods, and measured outcomes—a meta-analysis could not be conducted. Consequently, a descriptive approach was employed for data analysis. This review is not eligible for inclusion in PROSPERO because it completed data extraction; thus, registration information is not applicable.

### 2.7. Limitations of Included Evidence

Given that this review is based exclusively on case reports, several limitations must be acknowledged. Case reports are inherently subject to reporting bias, as they often describe unusual or severe presentations and may lack comprehensive or standardized clinical data. The heterogeneity in reporting styles, clinical detail, and outcome measures limits the ability to synthesize findings quantitatively. Furthermore, many reports lack long-term follow-up, making it difficult to assess the true efficacy of interventions or the incidence of complications.

## 3. Results

### 3.1. Literature Search

A total of 56 studies were identified through comprehensive database searches. Following the removal of duplicates, 20 studies remained, with 1 study excluded based on abstract evaluation. Ultimately, 19 reported cases of PID in non-sexually active pediatric and adolescent patients up to March 2025 were included in the analysis. A comprehensive flowchart for the selection of study is depicted in Figure 1.

### 3.2. Bias Risk Evaluation

To assess the risk of bias and ensure the methodological quality of the included case reports, each study was evaluated using the Joanna Briggs Institute (JBI) Critical Appraisal Checklist for Case Reports. This checklist consists of eight key criteria: (1) clear description of the patient’s demographic characteristics; (2) detailed history and timeline of the patient’s condition; (3) comprehensive description of the clinical condition at presentation; (4) clear reporting of diagnostic tests or assessment methods and results; (5) explicit details of the intervention or treatment procedure; (6) post-intervention clinical condition described clearly; (7) identification and description of adverse events or unanticipated outcomes; and (8) provision of takeaway lessons or conclusions.

Out of the total included case reports, 18 fulfilled at least seven out of eight criteria, indicating a generally high methodological quality. Most studies clearly described patient demographics, interventions, and outcomes, though several lacked explicit statements regarding adverse events or long-term follow-up. Any discrepancies in scoring between reviewers were resolved through discussion to ensure consistency. A summary of the quality-assessment results is provided in Table 1.

### 3.3. Studies Characteristics

A total of 20 case reports documented PID in non-sexually active pediatric and adolescent patients encompassing 22 patients aged between 11 and 17 years were analyzed. The demographic distribution revealed a predominance in early to mid-adolescence. Presenting symptoms exhibited variability, the predominant clinical symptom being lower abdominal pain, observed in 95.8% (21 out of 22 cases) of the cases. Fever was noted in 63.6% (14 cases), and gastrointestinal symptoms such as nausea, vomiting, diarrhea, constipation, or anorexia were reported in 50% (11 cases) of the cases. Dysuria was documented in 27.2% (six cases) and vaginal discharge in 9% (two cases) of the cases. Additionally, isolated presentations such as purpuric rash and macroscopic hematuria occurred in one patient (Table 2).

Several comorbid conditions were identified among the patients. Appendicitis history or post-appendectomy states were noted in 18.18% cases (four patients). Obesity was reported in 18.18% (four patients), while diabetes mellitus (type 1 or type 2) was present in 9% (two patients). Inflammatory bowel disease was seen in 9% (two patients), and congenital anomalies (including Hirschsprung disease or uterine anomalies) were documented in 18.18% (four patients). Urinary tract infections (UTIs) or recurrent UTIs were present in 22.72% (five patients). Chronic constipation appeared in 13.63% (three patients). Other individual conditions included gastroenteritis and Henoch–Schönlein purpura (Table 2).

Diagnostic imaging modalities primarily consisted of ultrasound (US) and computed tomography (CT). US was utilized in 86.36% of cases, while CT was utilized in 54.55% of cases. Findings from US and CT commonly revealed complex adnexal masses (40.91%, 9 cases), pyosalpinx (27.27%, 6 cases), and TOA (68.18%, 15 cases). Magnetic resonance imaging (MRI) was employed in a limited number of cases (13.64%, n = 3) for further pelvic pathology characterization (Table 2).

Microbiological cultures identified causative organisms in most cases. *Escherichia coli* was isolated in 27.2% (six patients) *Streptococcus* species (*viridans*, *anginosus*, *milleri*, *constellatus*, and *beta-hemolytic group F*) in 31.8% (seven patients), while anaerobes, including *Peptostreptococcus* and *Bacteroides* species, were isolated in 18.1% (four patients) of the cases. Other pathogens, such as *Fusobacterium nucleatum*, *Morganella morganii*, and *Staphylococcus aureus*, were found in isolated cases. Negative cultures were reported in twi patients (9%) (Table 3).

All 23 patients received broad-spectrum antibiotic therapy as part of their treatment regimen. Surgical intervention was necessary in 90.9% (20 out of 22) of cases. Surgical approaches included exploratory laparotomy in 36.4% (8 cases) and diagnostic laparoscopy in 59.1% (13 cases). Additionally, abscess drainage via interventional radiology was performed in one case. Salpingectomy, either partial or complete, was carried out in 36.4% (eight cases), while salpingo-oophorectomy was performed in 18.2% (four cases). Overall, abscess drainage—achieved through laparoscopy, laparotomy, or interventional radiology—was reported in 63.6% (14 cases) (Table 3).

Follow-up data revealed favorable outcomes in 72.7% (16 cases), with resolution of symptoms and infection. However, 27.2% (6 cases) experienced complications, including recurrence of TOA, persistent hydrosalpinx, sepsis, or postoperative infections. No mortalities were reported (Table 3).

## 4. Discussion

This systematic review highlights the occurrence of PID in non-sexually active pediatric and adolescent patients—an underrecognized yet clinically significant entity.

An increasing number of cases of pelvic inflammatory disease in pediatric patients have been reported in the literature; however, it remains a rare condition.

PID is typically associated with sexually transmitted infections; however, in sexually inactive pediatric patients, alternative non-sexually transmitted mechanisms must be considered. Several factors may predispose this population to PID, including hematogenous dissemination of pathogens, such as *Mycobacterium tuberculosis* in genital tuberculosis or *Streptococcus* species in cases of systemic sepsis, which can lead to upper genital tract infections. Direct extension of infections from neighboring organs, like appendicitis, peritonitis, and diverticulitis, has also been reported as a cause of PID in non-sexually active individuals [23,26]. Additionally, congenital reproductive tract anomalies, such as Müllerian anomalies or hydrocolpos, may create an environment conductive to ascending infections [6,7,17]. Patients with underlying immune deficiencies (e.g., primary immunodeficiencies, diabetes mellitus, and cystic fibrosis) are at a higher risk of developing PID due to impaired host defenses [6]. Furthermore, recent abdominal or pelvic surgeries, invasive gynecological procedures, and pelvic trauma can facilitate bacterial entry into the reproductive tract [6,18,19]. These findings suggest that PID in this population is more likely to be secondary to hematogenous spread or contiguous infection from neighboring pelvic or gastrointestinal structures than to ascending infection, the mechanism typically seen in sexually active individuals. Furthermore, the frequent association of PID with congenital genitourinary malformations—such as Müllerian anomalies and obstructive outflow tract defects—underscores the need for clinicians to maintain a high index of suspicion for PID even in sexually inactive pediatric patients, particularly when anatomical abnormalities are present.

The heterogeneity of clinical presentation across cases complicates early diagnosis. Although lower abdominal pain was nearly universal, associated symptoms were variable and often nonspecific, frequently overlapping with common pediatric conditions like appendicitis or urinary tract infections. Some patients may be asymptomatic or show only mild signs, while others present with severe, acute illness. The underreporting of vaginal discharge and the infeasibility of bimanual exams in virginal patients further limits the utility of traditional CDC diagnostic criteria in this demographic. This calls for refinement of diagnostic frameworks and the development of age- and context-specific guidelines.

Documented cases in the literature suggest that the differential diagnosis for pelvic inflammatory disease in sexually inactive pediatric patients is extensive, including conditions such as appendicitis, ovarian torsion, complications related to ovarian cysts, urinary tract infections, and gastrointestinal disorders like mesenteric adenitis and inflammatory bowel disease. The differential diagnosis of TOA includes ovarian cysts, dermoid cysts, other benign ovarian tumors, ovarian cancer, and ectopic pregnancy [1,2,14]. In the reviewed cases, appendicitis and urinary tract infections were the most common associated conditions. These overlapping clinical presentations may complicate the diagnostic process, potentially leading to misdiagnosis and delayed initiation of appropriate treatment for PID.

Diagnosing PID in virgin pediatric patients is challenging due to its atypical presentation and the absence of sexually transmitted infections as a primary cause. In sexually inactive patients, where bimanual pelvic examination may not be feasible, the diagnosis of PID often relies on surrogate findings, such as lower abdominal tenderness or fever. Laboratory findings may reveal leukocytosis, elevated C-reactive protein (CRP), and increased erythrocyte sedimentation rate (ESR), all indicative of systemic inflammation. Although CDC guidelines recommend clinical criteria such as cervical motion tenderness, uterine tenderness, or adnexal tenderness, alternative diagnostic approaches are essential in virgin patients to avoid invasive examination. Furthermore, although abnormal cervical or vaginal discharge is included in the CDC’s supportive diagnostic criteria for PID, these findings may be absent or not assessable in sexually inactive patients. In this review, abnormal discharge was reported in only two cases, highlighting the limited diagnostic utility of this criterion in this specific population. Due to the nonspecific clinical presentation of PID, ongoing research is exploring the use of noninvasive biomarkers to aid in the early identification of upper genital tract inflammation [11]. Several studies in the literature have reported a correlation between CA-125 levels and tubo-ovarian abscesses, suggesting that elevated CA-125 may reflect the severity of peritoneal inflammation rather than malignancy and might even serve as a marker for the failure of conservative treatment [33]. HIV testing is not routinely necessary in all non-sexually active pediatric PID cases due to their lower risk of HIV infection. However, testing should be considered when risk factors such as unclear PID etiology, history of sexual abuse, perinatal HIV exposure, or prior blood transfusions are present. Current guidelines also recommend routine HIV screening in adolescents aged 15 and older, regardless of sexual activity, to support early detection and intervention [13]. Pregnancy testing is essential in all PID cases to exclude pregnancy-related complications, even in sexually inactive patients. STI testing should also be considered based on clinical suspicion, especially in cases of unclear sexual history or potential abuse, to ensure appropriate management and antibiotic selection [3]. While CT and MRI are commonly used to detect TOA, evidence supporting the diagnostic accuracy of ultrasound is limited, with smaller studies suggesting sensitivities of 75% for transabdominal and 83% for transvaginal scans, though in their findings, only a minority of imaging studies explicitly identified TOA or pyosalpinx. In a large case series, Hakim et al. [4] reported that despite all patients ultimately being diagnosed with TOA, only 40% of transabdominal ultrasounds and 23% of CT or MRI scans specifically identified TOAs or pyosalpinx. In the reviewed case reports, ultrasound and CT were the main imaging modalities utilized for diagnosing PID, successfully detecting key features such as TOAs and pyosalpinx in most cases. Although MRI offers superior tissue characterization, it was used infrequently, likely due to accessibility and the adequacy of other imaging methods.

While most guidelines, including those from the CDC, emphasize empiric antibiotic treatment for sexually active individuals, sexually inactive pediatric patients diagnosed with PID should still receive broad-spectrum antimicrobial therapy. Antibiotic regimens applied in the evaluated studies were mostly adherent to CDC treatment guidelines [34]. This is due to the polymicrobial nature of the infection, which often involves enteric organisms, anaerobes, and respiratory flora rather than sexually transmitted pathogens. Recommended treatment regimens may mirror those for sexually active patients but should particularly target organisms such as *Escherichia coli*, *Streptococcus* species, and anaerobes commonly implicated in non-sexually transmitted cases. However, few studies provided follow-up data on antimicrobial resistance patterns or recurrence rates, revealing an area needing further investigation. The Pelvic Inflammatory Disease Evaluation and Clinical Health (PEACH) trial evaluated the efficacy of outpatient versus inpatient treatment strategies for female patients with mild-to-moderate PID [35]. The trial concluded that outpatient management with appropriate antibiotic therapy is as effective as inpatient care in preventing long-term reproductive complications, such as infertility and chronic pelvic pain. This finding is particularly relevant for adolescents, supporting the use of outpatient treatment protocols in clinically stable patients, provided there is adequate follow-up and adherence to therapy. In their study, Trent et al. [13] conducted a retrospective review of medical records from 56 adolescent patients diagnosed with pelvic inflammatory disease (PID) in pediatric outpatient settings. The study revealed that 40% of these patients were prescribed inadequate medication regimens. Notably, those treated in pediatric emergency departments were less likely to receive standard medication protocols compared to those seen in ambulatory settings. Additionally, the majority of patients did not receive sufficient self-care instructions upon discharge, irrespective of the care location. In the reviewed case reports, empirical antibiotic treatment was generally effective, and a high proportion of cases (over 90%) required surgical intervention, often due to advanced disease at presentation. This raises concern about diagnostic delays and highlights the need for heightened clinical suspicion in evaluating pediatric abdominal pain. Supportive care, including pain management and fluid support, is essential in the overall treatment strategy [36].

In cases of TOA or pyosalpinx, where the clinical course deteriorates or diagnostic uncertainty persists, surgical intervention, and diagnostic and therapeutic laparoscopy offer the dual benefits of confirming the diagnosis and allowing for abscess drainage, debridement, and adhesion lysis—all with a focus on preserving reproductive anatomy [36]. Although traditional surgical intervention, particularly incision and drainage, remains the standard of care in the management of TOA, IR-guided drainage has increasingly been explored as a minimally invasive alternative [4]. Since prior surgical intervention is a known predisposing factor for noncommunicable pyosalpinx, interventional radiology may serve as a beneficial minimally invasive option, particularly for patients at higher surgical risk or where fertility preservation is a priority [37]. Ultrasound- or CT-guided transabdominal or transrectal approached aspiration can be considered in hemodynamically stable patients with well-encapsulated abscesses who do not respond to antibiotics alone. However, the data supporting its routine use in pediatric and adolescent populations are limited. The technique, while promising, should currently be viewed as a complement rather than a replacement for surgical management, particularly given the anatomical and clinical complexities in this age group. The primary risks associated with image-guided drainage of pyosalpinx and TOA include access-site infection, hemorrhage, pelvic organ injury, and procedure-related pain [37]. Further studies are needed to establish clear indications, safety, and long-term outcomes of IR-guided interventions in this setting [10].

A critical gap in the current literature is the paucity of long-term outcomes. Early intervention with broad-spectrum antibiotics, often complemented by minimally invasive surgical techniques such as laparoscopy or image-guided drainage, tends to result in resolution of the inflammatory process and minimizes complications [12]. However, delays in diagnosis or treatment can increase the risk of complications. Short-term complications of PID can include perihepatitis, known as Fitz–Hugh–Curtis syndrome, as well as peri-appendicitis. Although the exact mechanism behind perihepatitis remains uncertain, it occurs in up to 15% of PID cases. Clinically, patients often present with varying degrees of right upper quadrant abdominal pain, accompanied by tenderness, guarding, and occasionally mild hepatomegaly upon examination [3]. Patients diagnosed with PID face elevated risks of ectopic pregnancy, infertility, and chronic pelvic pain, largely due to tubal scarring and inflammatory damage. Infection in PID induces fibrinous or purulent inflammation along the fallopian tube and ovarian surfaces, often resulting in scarring, adhesions, and partial or complete tubal obstruction. Damage to the ciliated epithelial cells of the fallopian tubes impairs ovum transport, increasing the risk of tubal-factor infertility and ectopic pregnancy. Additionally, peritoneal adhesions contribute to infertility and are associated with chronic pelvic pain [4]. Data from the PEACH trial indicated that 36% of participants experienced chronic pelvic pain, with the highest risk observed in women who had multiple PID episodes [5]. The reviewed case reports documented several complications associated with PID in pediatric patients. In the follow-up period, some patients experienced recurrence of TOA. Additionally, persistent hydrosalpinx was observed in some cases. Other complications reported in the case reports included sepsis, wound infection, and recurrent intra-abdominal abscess. Collectively, these complications underscore the critical importance of early diagnosis and prompt, effective management of PID [38,39].

The findings of this review are limited by the nature of the included evidence, which consists exclusively of case reports. While case reports are valuable for identifying rare or atypical presentations, they often lack standardized reporting, are susceptible to publication and selection bias, and may not be generalizable to broader clinical populations. There is a strong tendency to publish more severe or surgically managed cases, which likely underrepresents milder or conservatively treated instances of PID. Moreover, many case reports provide limited clinical details and often omit long-term follow-up, restricting the ability to assess treatment efficacy and complication rates over time. The absence of controlled or comparative studies further limits the capacity to draw definitive conclusions about causality, optimal therapeutic strategies, or prognostic factors. Additionally, heterogeneity in reporting styles, diagnostic criteria, and outcome measures hinders the feasibility of data synthesis or meta-analysis.

Future research should prioritize prospective data collection, ideally through multicenter registries or cohort studies, and focus on developing noninvasive diagnostic biomarkers for early identification. There is also a pressing need for standardized, age-appropriate diagnostic criteria and management algorithms tailored specifically to sexually inactive pediatric and adolescent populations, to ensure timely and effective care while preserving reproductive health.

## 5. Conclusions

Pelvic inflammatory disease presents significant challenges in women’s health, often resulting in complications such as TOA and infertility. While commonly associated with sexually active women, it should also be considered in the differential diagnosis of abdominal pain and pelvic masses in pediatric and adolescent females. This review highlights the importance of recognizing PID in children and adolescents, regardless of sexual history, to ensure timely diagnosis and treatment. Further research is needed to better define prognostic factors, optimize diagnostic strategies, and refine long-term management approaches for PID in pediatric and adolescent populations.

## Figures and Tables

**Table 1:** Quality assessment according to JBI Critical Appraisal Tool: Checklist for Case Reports.

Reference	1	2	3	4	5	6	7	8	Total	Quality
Moralioğlu et al. [7]	Y	Y	Y	N	N	N	Y	Y	5/8	Moderate
Simpson-Camp et al. [17]	Y	Y	N	Y	Y	Y	Y	Y	7/8	High
McKinnon et al. [6]	Y	N	Y	Y	Y	Y	Y	Y	7/8	High
Campbell et al. [18]	Y	Y	Y	Y	Y	Y	U	Y	7/8	High
Goodwin et al. [19]	Y	Y	Y	Y	Y	Y	Y	Y	8/8	High
Arda et al. [8]	Y	Y	Y	Y	Y	Y	U	Y	7/8	High
Hartmann et al. [20]	Y	Y	Y	Y	Y	Y	U	Y	8/8	High
Rubino et al. [21]	Y	Y	Y	N	Y	Y	Y	Y	7/8	High
Stortini et al. [22]	Y	Y	Y	Y	Y	Y	U	Y	7/8	High
Nishida et al. [23]	Y	Y	Y	Y	Y	Y	Y	Y	8/8	High
Moore et al. [24]	Y	Y	Y	Y	Y	Y	Y	Y	8/8	High
Pomeranz et al. [25]	Y	Y	Y	Y	Y	Y	U	Y	7/8	High
Mills et al. [26]	Y	Y	Y	Y	Y	Y	U	Y	7/8	High
Cheong et al. [27]	Y	Y	Y	Y	Y	Y	Y	Y	8/8	High
Fink et al. [5]	Y	Y	Y	Y	Y	Y	Y	Y	8/8	High
Boleken et al. [28]	Y	Y	Y	Y	Y	Y	Y	Y	8/8	High
Algren et al. [29]	Y	Y	Y	Y	Y	Y	Y	Y	8/8	High
Kielly et al. [30]	Y	Y	Y	Y	Y	Y	Y	Y	8/8	High
Murata et al. [31]	Y	Y	Y	Y	Y	Y	Y	Y	8/8	High
Sakar et al. [32]	Y	Y	N	Y	Y	Y	Y	Y	6/8	Moderate

Key: Y, yes; N, no; U, unknown. (1) Clear description of the patient’s demographic characteristics; (2) detailed history and timeline of the patient’s condition; (3) comprehensive description of the clinical condition at presentation; (4) clear reporting of diagnostic tests or assessment methods and results; (5) explicit details of the intervention or treatment procedure; (6) post-intervention clinical condition described clearly; (7) identification and description of adverse events or unanticipated outcomes; and (8) provision of takeaway lessons or conclusions.

**Table 2:** Summary of patient demographics, clinical presentation, coexisting conditions, and imaging findings.

Reference	Age (Years)	Diagnosis	Clinical Presentation	Coexisting Conditions	Imaging
Moralioğlu et al. [7]	13	Right-sided hydrosalpinx	Abdominal pain	Hirschsprung disease (HD)	CT scan: septated cyst measuring 10 × 6 × 7 cm^3^ in size
Moralioğlu et al. [7]	14	Right-sided pyosalpinx	Abdominal pain, vomiting, fever	Rectovestibular fistula, anal atresia, sigmoid resection, uterus bicornis unicollis, septate vagina	Ultrasound scan: cystic lesion (10.5 cm × 7.5 cm) with internal septations in the right adnexal region
Simpson-Camp et al. [17]	14	Left TOA	Fatigue, fever, pelvic pain, abdominal fullness, dysuria	Not mentioned	Ultrasound scan: complex right adnexal mass measuring 12.5 cm/9.6 cm/11 cm with multicystic areas and septations
McKinnon et al. [6]	13	Bilateral pyosalpinx	Nausea, vomiting, fever, diffuse abdominal and pelvic pain	Obesity, asthma, type 1 diabetes mellitus	Ultrasound scan: left ovarian cyst (4.38 × 3.42 cm^2^) with smooth contours, reduced venous Doppler flow, moderate amount of free fluid
Campbell et al. [18]	15	Right pyosalpinx	Abdominal pain	Recent appendectomy postoperative peritonitis	Ultrasound scan: right annexation lesion adjacent to the right ovary measuring 7.1 cm × 4.3 cm × 4.3 cm
Goodwin et al. [19]	13	Bilateral TOA	Abdominal pain, vomiting	Constipation	Abdominal radiography: air fluid levels US scan: intestinal occlusion and potential perforation
Arda et al. [8]	15	Right TOA	Right lower abdominal pain, dysuria, fever	Urinary tract infections	US and CT scan: 6 × 2.5 × 3 cm^3^ abscess originating in the right tubo-ovarian structures
Hartmann et al. [20]	16	Right TOA	Abdominal pain in the right lower quadrant, fever	Inflammatory bowel disease, Candida vaginitis	US scan: small right ovarian cyst CT scan: small irregular fluid collections extending into the pelvis, anterior and superior to the uterus with inflammation of the right ovary
Hartmann et al. [20]	12	Bilateral TOA	Diffuse lower abdomen pain, nausea, vomiting, fever	Obesity, type 2 diabetes mellitus, constipation, recurrent UTI	CT scan: echogenic debris at the center of the lower pelvis, suggestive of large dominant cyst and inflammation of the left ovary
Rubino et al. [21]	16	Bilateral salpingitis	Lower quadrant abdominal pain, fever, leukorrhea	Chronic appendicitis	MRI scan: bilateral salpingitis
Stortini et al. [22]	14	Bilateral TOA	Acute urinary retention	Lichen sclerosus Recurrent UTI	MRI scan: severe inflammatory changes of the pelvis, TOAs mainly in the right ovary and both fallopian tubes
Nishida et al. [23]	15	Right TOA	Recurrent fever, right lower quadrant pain	Appendectomy	CT scan: cystic structures with thickened walls in the right pelvis
Moore et al. [24]	15	Left TOA	Abdominal pain, nausea, vomiting, dysuria and fever	Obesity, cystitis	US scan: enlarged heterogenous uterus, small fluid collection in the fundus MRI scan: abovementioned findings, poorly defined soft tissue changes
Pomeranz et al. [25]	15	Left TOA	Abdominal pain, vomiting, purpuric rash, hematuria	Recurrent episodes of Henoch–Schönlein purpura	US scan; multiloculated mass localized to the left ovary CT scan: left semisolid ovarian mass
Mills et al. [26]	13	Bilateral tubo-ovarian abscess	Intermittent abdominal pain for several months	Appendicitis	CT scan: 5.2 × 5.8 × 5.3-cm^3^ multiloculated cystic mass with surrounding inflammation and adjacent peripherally enhancing fluid
Cheong et al. [27]	13	Left-sided pyosalpinx	Fever, anorexia, vomiting, abdominal pain, vaginal discharge	Peritonitis	US scan: retrouterine heterogenous collection measuring 10.8 × 11.4 cm^2^ that was compatible with an abscess secondary to perforated appendicitis
Fink et al. [5]	11	Left tubo-ovarian abscess	Left lower abdominal pain, blood-streaked emesis, anorexia	Enuresis, obesity	CT scan: Lower abdominal mesentery heterogenous complex lesion MRI scan: hydrosalpinx with thick peripheral enhancement
Boleken et al. [28]	15	Left-sided pyosalpinx	Left lower quadrant abdominal pain	Chronic constipation	Ultrasound scan: thick-walled, dense cystic mass of 10 × 10 cm^2^ CT scan: 9.3 × 10 × 11 cm^3^ cystic lesion, suggesting a pyosalpinx
Algren et al. [29]	14	Bilateral hydrosalpinges; right TOA	Abdominal pain, dysuria, nausea, vomiting, diarrhea, weight loss, fevers, night sweats, fatigue	Gastroenteritis	US scan: large complex (mostly solid) pelvic mass of 10.8 × 7.9 × 9.9 cm^3^. CT scan: multiloculated fluid collection
Kielly et al. [30]	15	Pelvic inflammatory disease	General malaise, diarrhea, right lower quadrant pain.	Fitz–Hugh–Curtis syndrome	Ultrasound and CT scan: moderate amount of free fluid in the pelvis and right lower quadrant
Murata et al. [31]	13	Right TOA	Fever	None	Ultrasound scan: pelvic mass measuring 6 cm CT scan: unilateral and unilocular ovarian mass
Sakar et al. [32]	13	Subacute salpingitis, Left TOA	Abdominal pain, menstrual disorder	None	US scan: semisolid, hyperechogenic mass of 57 × 73 mm^2^ in the left adnexal area CT scan: dense cystic semisolid mass (7 × 6.4 cm^2^) with thickened walls and peripheral contrast

TOA, tubo-ovarian abscess; UTIs, urinary tract infections; US, ultrasound; CT, computed tomography; MRI, magnetic resonance imaging.

**Table 3:** Summary of therapeutic interventions, microbiological findings, and clinical outcomes.

Reference	Surgical Management	Microorganism Cultured	Antibiotic Regimens	Follow-Up/ Recurrence
Moralioğlu et al. [7]	Exploratory laparotomy: right salpingectomy	Not mentioned	Not mentioned	No further complications
Moralioğlu et al. [7]	Exploratory laparotomy: right salpingectomy	*Escherichia coli*	Inpatient: IV ceftriaxone/metronidazole	No further complications
Simpson-Camp et al. [17]	Exploratory laparotomy: abscess drainage	*Streptococcus* *viridans*	Preoperatively: IV cefazolin (single doses) Postoperatively: IV doxycycline/cefoxitin changed to cefotaxime × 14 days	Superficial fluid collection at the inferior portion of her wound developed on 30th post-operative day
McKinnon et al. [6]	Diagnostic laparoscopy: bilateral salpingostomies, drainage	*Fusobacterium* *nucleatum*	Inpatient: IV cefoxitin/doxycycline × 10 days Outpatient: PO metronidazole × 1 month	Resolution of symptoms on day 1 postoperatively
Campbell et al. [18]	Diagnostic laparoscopy: abscess drainage	Negative	Inpatient: IV doxycycline/metronidazole/cefoxitin	No further complications
Goodwin et al. [19].	Exploratory laparotomy: abscess drainage	Ampicillin-sensitive *Escherichia coli*	Preoperatively: IV gentamycin (5 mg/kg)/metronidazole (500 mg). Postoperatively: IV clindamycin (40 mg/kg/d)/gentamycin (5 mg/kg/d)	Complete resolution of bilateral TOA on ultrasound scan at 3 months
Arda et al. [8]	Diagnostic laparoscopy: Abscess drainage	*Escherichia coli*	Inpatient: IV ceftriaxone (100 mg/kg, 24 h)/amikacin (15 mg/kg, 12 h)	No further complications
Hartmann et al. [20]	Diagnostic laparoscopy	*Bacteroides uniformis*, *Coagulase negative Staphylococcus*, *Streptococcus milleri*	Inpatient: IV doxycycline/gentamycin/cefotaxime/metronidazole doxycycline Outpatient: PO Doxycycline/Metronidazole × 14 days	No further complications
Hartmann et al. [20]	Diagnostic laparoscopy	*Escherichia coli*	Inpatient: IV doxycycline/gentamycin/cefotaxime/metronidazole Outpatient: PO doxycycline/metronidazole × 14 days	Persistence of hydrosalpinx
Rubino et al. [21]	Diagnostic laparoscopy: adhesiolysis; appendectomy	Not mentioned	Inpatient: IV ceftriaxone/metronidazole Outpatient: PO azithromycin × 14 days	Persistence of hydrosalpinx
Stortini et al. [22]	Abscess drainage by interventional radiology	*Streptococcus* *anginosus* *Peptostreptococcus anaerobius*	Inpatient: IV tobramycin (7.5 mg/kg/d)/metronidazole (30 mg/kg/d) × 14 days Outpatient: PO amoxicillin/clavulanic acid (1500 mg/d) × 10 days	No further complications
Nishida et al. [23]	Diagnostic laparoscopy: abscess drainage	Negative	Inpatient: IV Cefmetazole	Recurrence of TOA after 2 months
Moore et al. [24]	Diagnostic laparoscopy, exploratory laparotomy: left salpingo-oophorectomy	*Escherichia coli*	Preoperatively: IV ceftazidime for pyelonephritis	Sepsis, wound infection Recurrent intra-abdominal abscess
Pomeranz et al. [25]	Exploratory laparotomy: left salpingo-oophorectomy	*Morganella morganii*	Inpatient: IV ampicillin/gentamicin/metronidazole	No further complications
Mills et al. [26]	Exploratory laparotomy: abscess drainage	*Streptococcus constellatus*	Inpatient: IV piperacillin/tazobactam × 12 days	No further complications
Cheong et al. [27]	Diagnostic laparoscopy: left salpingectomy	*Streptococcus viridans* *Peptostreptococcus*	Inpatient: IV piperacillin/tazobactam × 5 days Outpatient: PO amoxicillin/clavulanic acid × 14 days	Resolution of bilateral TOA on ultrasound scan at 2 months
Fink et al. [5]	Diagnostic laparoscopy: abscess drainage;	*Streptococcus* *bovi*, *Bacteroides thetaiotaomicron*	Inpatient: cefoxitin (2000 mg)/doxycycline (100 mg), ampicillin/sulbactam (2000 mg) Outpatient: PO amoxicillin/clavulanic × 14 days	Recurrence of left tubo-ovarian abscess after 1 month
Boleken et al. [28]	Exploratory laparotomy: left salpingectomy	*Escherichia coli*	Inpatient: IV broad-spectrum as preoperative preparation Outpatient: PO meropenem (40 mg/kg/d)	Resolution of symptoms after 2 days postoperatively
Algren et al. [29]	Diagnostic laparoscopy, exploratory laparotomy: right salpingo-oophorectomy	*Beta Hemolytic Streptococcus* *Group F*	Ampicillin, gentamycin, and Clindamycin (preoperatively); metronidazole and ceftriaxone (postoperatively) later changed to clindamycin and ciprofloxacin	Resolution on CT scan after 1 month
Kielly et al. [30]	Diagnostic laparoscopy	Not mentioned	Preoperatively: ceftriaxone 2 g intravenously (IV), metronidazole 500 mg IV, and vancomycin 1 g IV Postoperatively: ceftriaxone 2 g IV × 24 h, metronidazole 500 mg IV × 8 h, and doxycycline 100 mg IV × 12 h	Suspected pulmonary embolism; Severe secondary dysmenorrhea Recurrence 1 year post-operatively
Murata et al. [31]	Diagnostic laparoscopy: right salpingo-oophorectomy	*Methicillin-susceptible*, *Staphylococcus aureus*	Inpatient: IV cefmetazole for 5 days, 2 g/day Outpatient: PO cefaclor at a dose of 900 mg/day × 14 days	Resolution on MRI scan after 1 month
Sakar et al. [32]	Exploratory laparotomy: Abscess drainage	Not mentioned	Inpatient: IV ceftriaxone (2 g/day)/metronidazole (500 mg/day) Outpatient: PO metronidazole/cefuroxime × 14 days	Resolution of symptoms after 7 days postoperatively

IV, intravenously; PO, orally.

## Data Availability

Data sharing is not applicable, since the article is a review of the literature.

## Abbreviations

The following abbreviations are used in this manuscript:

PDI	Pelvic inflammatory disease
TOA	Tubo-ovarian abscess
US	Ultrasound
CT	Computed tomography
MRI	Magnetic resonance imaging
IV	Intravenously
PO	Orally
